# Coronary artery calcification and dietary intake in asymptomatic men

**DOI:** 10.1590/1414-431X2021e11371

**Published:** 2021-09-20

**Authors:** N.M. Bruscato, P.L. da Luz, B.M. Werle, P.R. Schvartzman, J. Kesties, L. Vivian, W. de Carli, E.H. Moriguchi

**Affiliations:** 1 Programa de Pós-Graduação em Ciências da Saúde: Cardiologia e Ciências Cardiovasculares, Universidade Federal do Rio Grande do Sul, Porto Alegre, RS, Brasil; 2 Instituto do Coração, Hospital das Clínicas, Faculdade de Medicina, Universidade de São Paulo, São Paulo, SP, Brasil; 3 Serviço de Radiologia, Hospital Moinhos de Vento, Porto Alegre, RS, Brasil; 4 Associação Veranense de Assistência em Saúde, Veranópolis, RS, Brasil; 5 Departamento de Pesquisa, Instituto Moriguchi: Centro de Estudos do Envelhecimento, Veranópolis, RS, Brasil; 6 Departamento de Medicina Interna, Hospital de Clínicas de Porto Alegre, Universidade Federal do Rio Grande do Sul, Porto Alegre, RS, Brasil

**Keywords:** Vascular calcification, Diet, Lipids, Fatty acids, Carbohydrates, Men

## Abstract

Dietary factors may influence the process of atherosclerosis and coronary artery calcification (CAC). This study assessed CAC and its association with dietary intake in asymptomatic men. We evaluated 150 asymptomatic men with mean age of 58.2±5.3 years. The dietary intake was assessed by the Food Consumption Register method. CAC was measured through multidetector computed tomography (MDCT) and assessed in accordance with the Agatston score. Modified Poisson regression model was used to estimate the effects of intake of different nutrients that are prevalent in moderate/severe CAC, adjusted for calorie intake and CAC risk factors by means of prevalence ratios and 95% confidence intervals [95%CI]. An association was found between the intake of some nutrients and moderate/severe CAC. Lower carbohydrate intake (P=0.021) and higher lipid intake (P=0.006) were associated with moderate/severe CAC. After adjustment, the nutrients associated with the prevalence of moderate/severe CAC were carbohydrates (P=0.040), lipids (P=0.005), and saturated fatty acids (SFA) (P=0.013). A 1% increase in lipids and SFA intake caused an increase of 4% [95%CI: 1-7%] and 8% [95%CI: 2-14%] in the prevalence of moderate/severe CAC, respectively. A 1% increase of carbohydrate intake led to a 2% decrease in the likelihood of moderate/severe CAC [95%CI: 1-4%]. These conclusions showed that the higher intake of total lipids and SFA was associated with higher CAC scores, whereas higher carbohydrate intake was associated with lower CAC scores in asymptomatic men.

## Introduction

Coronary artery disease (CAD) is the main cause of death in the USA and is the most common of cardiovascular diseases, accounting for 1 in 7 deaths in the USA (1). One of the main causes of CAD is atherosclerosis, a chronic, inflammatory disease that affects the intima of large- and medium-caliber arteries (2). Coronary atherosclerosis starts in a subclinical stage and slowly progresses over the years prior to the development of cardiovascular events (3). Coronary artery calcification (CAC) is marked by calcium deposits in the atherosclerotic plaque of the coronary artery wall and is considered a specific component of coronary atherosclerosis (4). Arterial calcification is a complex phenomenon and generally considered to be a late event in the progression of the atherosclerotic plaque (5).

Studies show that risk factors such as age, sex, family history of CAD, high blood pressure, high cholesterol levels, diabetes, smoking, obesity, and sedentary lifestyle are associated with CAC (6-13). Regarding nutrition, there are few studies addressing its relationship with CAC. Recent studies have shown no association between the composition of dietary macronutrient intake and CAC (14,15). Only one study has shown association between saturated fatty acid (SFA) intake and carbohydrate intake with CAC (16). Other studies showed an inverse association of the nutrients linolenic acid (17) and magnesium with CAC (18).

Due to the scarcity of information available about the role of dietary factors in the development of CAC, we investigated the association of macronutrients and mineral micronutrients calcium, magnesium, sodium, and potassium with CAC.

## Material and Methods

### Study population

The Study of Indexes of Aging and the Prevalence of Atherosclerosis in Habitual Wine Drinkers *vs* Abstainers is a population-based cross-sectional study. Its main objective is to evaluate indexes of arterial aging and the prevalence of atherosclerosis in habitual wine drinkers and in those who abstain from drinking. The São Paulo (SP) Heart Institute (Instituto do Coração de São Paulo, Brazil) developed the protocol. The original study took place in São Paulo (Brazil), with data on a complimentary sample collected in Veranópolis, Rio Grande do Sul (Brazil). A detailed description of the study design and methods has been published elsewhere (5).

The complementary study conducted with inhabitants of Veranópolis involved division of the sample based on habitual (rather than social) red wine consumption, as was conducted with the sample from São Paulo. The study involved 150 white men aged between 50-70 years who lived in Veranópolis. The relationship between red wine consumption and coronary calcium was previously reported (19) and showed that wine consumption was associated with higher coronary calcium but lower event rates.

Men with insulin-dependent diabetes mellitus, family dyslipidemia, neoplasms, liver, kidney, and/or heart dysfunction, known chronic coronary insufficiency, body mass index (BMI) <22 kg/m^2^ or >30kg/m, hypertension diagnosis (blood pressure >160/120 mmHg with or without anti-hypertensive drug use), depression diagnosis, those who smoked more than one pack of cigarettes/day, East Asian descendants, and/or consumed ≥50 g alcohol/day were excluded. Socioeconomic, demographic, anthropometric, biochemical, and dietary data were used for this study. The included variables were: age, sex, schooling, family income, weight, physical activity level, BMI, total cholesterol level, cholesterol fractions high- and low-density lipoproteins (HDL and LDL, respectively), triglycerides (TG), fasting blood glucose, blood pressure, alcohol intake, smoking and family history of premature CAD, daily intake of calories (kcal), macronutrients (total carbohydrates, proteins, and lipids), fatty acids (monounsaturated, polyunsaturated, and SFA), cholesterol, total fibers, sodium, potassium, magnesium, and calcium.

The Research Ethics Committee of the Hospital de Clínicas de Porto Alegre (HCPA), Porto Alegre, RS, Brazil approved this research (project number 130453).

### Cardiovascular risk factors and questionnaire

Data were collected using a structured questionnaire during the initial study (2011-2013) at the São Peregrino Lazziozi Community Hospital in Veranópolis, RS, Brazil. Blood samples were collected in the laboratory for the determination of lipid and glycemic profiles after a ≥12-h fast.

Educational level was divided into three categories: ≤8, 9-12, and >12 years. Income was classified as: <5, 5-10, or >10 times the minimum monthly wages in Brazilian real (1 USD=1.85 BRL).

Physical activity was classified as <150 or ≥150 min/week, following the guidelines of the American College of Sports Medicine and the American Heart Association, which recommend at least 30-min of moderate-intensity aerobic physical activity on 5 days/week or 20-min high-intensity aerobic physical activity on 3 days/week (20). A sedentary lifestyle was defined as <150 min/week exercise.

Subjects were classified as smokers and non-smokers. Participants reported regular consumption of alcohol on a daily basis during the clinical evaluation, and the amount of alcohol consumed was quantified (in grams) using the São Paulo Medical School's (Escola Paulista de Medicina) Nutwin^®^ Nutrition program (https://sourceforge.net).

Body weight (in kilograms) and height (in meters) were measured using a single mechanical anthropometric scale (Filizola^®^ SA, Brazil), previously inspected, with a fixed stadiometer. Abdominal circumference was determined halfway between the iliac crest and lower costal edge using a metric tape measure. BMI was calculated using the Quetelet index. Values <5 kg/m^2^ were considered to indicate normal weight, those of 25-30 kg/m^2^ were considered to indicate overweight, and values >30 kg/m^2^ were considered to indicate obesity, following the recommendations of the World Health Organization (21).

Hypertension was defined as systolic blood pressure ≥140 mmHg and/or diastolic blood pressure ≥90 mmHg and/or use of hypertensive medication (22).

Diabetes mellitus was defined as fasting glycemia ≥126 mg/dL or the use of hypoglycemic medications (23).

Dyslipidemia was defined following the V Brazilian Guidelines for Dyslipidemia and the Prevention of Atherosclerosis (24), which considers the benchmarks to be LDL-cholesterol ≥160 mg/dL, TG ≥150 mg/dL, and HDL-cholesterol <40 mg/dL for men or use of lipid-lowering agents.

Participants were considered to have a family history of premature CAD when their mothers and/or fathers, at an age below 55 for men or below 65 for women, has suffered fatal or non-fatal myocardial infarction and/or underwent coronary angioplasty or myocardial vascularization surgery (25).

### Measurement of coronary artery calcification

Coronary calcification was measured using a Siemens^®^ Somaton Sensation 64-detector CT scanner at the Moinhos de Vento Hospital in Porto Alegre, Brazil. Three-millimeter-thick slices were used for the calculation of calcium scores. CAC was evaluated using the Agatston score (26) and Cascoring software, at a Siemens^®^ workstation used specifically for coronary angiotomography. In the initial study, CAC scores (calculated using the Agatston method) were classified as 0, 1-10, 11-100, 101-400, and >400, representing very low, low, moderate, moderately high, and high cardiovascular risk, respectively (27). To facilitate data interpretation and the consideration of prevalence, CAC scores were classified as follows: no evidence (0), minimal CAC (1-10), moderate CAC (11-100), and severe CAC (>100). For this analysis, calcium scores were classified as ≤10 (no evidence and minimal CAC) and >10 (moderate and severe CAC).

### Assessment of dietary intake

For the dietary intake assessment, the Food Consumption Register method developed by Buzzard (28) was applied during three alternate days of the week, including one weekend day, by a duly trained dietician. According to this method, the respondents report all foods and drinks they had on those days, including those they had been away from home.

For nutrient calculation, the dietary software Nutwin^®^ developed by Escola Paulista de Medicina (Brazil) was used. Data on dietary intake included the total amount of calories (kcal), macronutrients (total carbohydrates, proteins, and lipids), fatty acids (monounsaturated, polyunsaturated, saturated), cholesterol, total fiber, sodium, potassium, magnesium, and calcium.

### Statistical analysis

Data analysis was performed using the SPSS software (SPSS Inc. PASW Statistics for Windows, Version 21.0, USA).

Continuous variables are reported as mean and standard deviation (SD) or median and interquartile range. Categorical variables are reported as absolute and relative frequencies. To compare the continuous variables between the groups, the Student's *t-*test was used. In case of asymmetry, the Mann-Whitney test was applied. For an adjusted calorie-intake combination, the covariance analysis (ANCOVA) was performed.

To control for confounding factors in evaluating the association of nutrients with CAC, a multivariate linear regression model was used. For variables with asymmetric distribution, logarithmic transformation was applied for the utilization of parametric tests.

The statistical significance level considered was 5% (P≤0.05).

## Results

The sample included 150 men with a mean age of 58.2±5.3 years. Sociodemographic, clinical, and laboratory features of the study subjects are presented in Table 1.

**Table 1:** Characteristics of the study subjects.

Variables	Total sample (n=150)
Age (years)	58.2±5.3
Education level (years)	
≤8	86 (57.3)
9-12	39 (26.0)
>12	25 (16.7)
Smoking (n, %)	
No	132 (88.0)
Yes	18 (12.0)
Average alcohol consumption (g/day)	28.7 (0.0-38.8)
Level of physical activity (min/week)	
<150	25 (16.7)
≥150	125 (83.3)
BMI (kg/m^2^)	26.8±2.5
Eutrophic (<25 kg/m^2^) (n, %)	44 (29.3)
Overweight (25-29.9 kg/m^2^) (n, %)	106 (70.7)
Waist (cm)	96.3±7.9
Normal	115 (76.7)
Enlarged (man >102)	35 (23.3)
Family history of CAD	23 (15.3)
Hypertension	96 (64.0)
Diabetes mellitus	14 (9.3)
Dyslipidemia	102 (68.0)
Use of statins	5 (3.3)
CAC	
No evidence	61 (40.7)
Minimal (1-10)	12 (8.0)
Moderate (11-100)	45 (30.0)
Severe (>100)	32 (21.3)

Quantitative data are reported as means±SD or median and percentiles (25th-75th) and categorical data as absolute (n) and relative (%) frequencies. CAD: coronary artery disease; CAC: coronary artery calcification.

Of note is the fact that 40.7% of the subjects had no coronary artery calcification (score=0). From those who presented coronary artery calcification (59.3%), 8% had mild CAC (score 1-10), 30% had moderate CAC (score 11-100), and 21.3% had severe CAC (score >100).

Table 2 presents descriptive statistics on the intake of calories, macronutrients, and micronutrients for the entire sample.

**Table 2:** Assessment of calorie, macronutrient, and micronutrient intake.

Variables	Total sample (n=150)
Calories (kcal)	2029±529
Carbohydrates (g/day)	254±86.29
Carbohydrates (%)	52.4±7.3
Proteins (g/day)	94.76±25.29
Proteins (%)	19.8±3.4
Lipids (g/day)	59.10±18.37
Lipids (%)	27.6±5.9
Fibers (g/day)	23.9 (16.8-33.1)
SFA (g/day)	18.2±6.9
SFA (%)	8.2±2.9
Polyunsaturated fatty acids (g/day)	12.2±4.7
Polyunsaturated fatty acids (%)	5.6±1.8
Monounsaturated fatty acids (g/day)	19.5±7.0
Monounsaturated fatty acids (%)	8.8±2.9
Cholesterol (mg/day)	261±104
Linoleic acid (g/day)	11.1±5.5
Linolenic acid (g/day)	1.28 (1.04-1.61)
Oleic acid (g/day)	17.6±6.8
Calcium (mg/day)	678.7±225
Magnesium (mg/day)	304.6±4.1
Potassium (mg/day)	3111±932
Sodium (mg/day)	4056±948

Quantitative data are reported as means±SD or median and percentiles (25th-75th). SFA: saturated fatty acids.

Table 3 presents the results of the association between dietary intake variables and pooled CAC (CAC ≤10 and CAC >10) from the gross bivariate and calorie-intake adjusted analysis. After calorie-intake adjustment, only lower carbohydrate intake (P=0.021) and higher lipid intake (P=0.006) presented statistically significant associations with moderate/severe CAC.

**Table 3:** Association between the intake of calories, macronutrients, and micronutrients with coronary artery calcification (CAC).

Variables	No evidence/minimal CAC (≤10) (n=73) Mean±SD [adjusted mean]	Moderate/severe CAC (>10) (n=77) Mean±SD [adjusted mean]	P**	Adjusted P^#^
Calories (kcal)	2114±581	1947±463	0.053	-
Carbohydrates (%)	54.1±7.4 [53.8]	50.8±7.0 [51.1]	**0.006**	**0.021**
Proteins (%)	19.5±3.6 [19.6]	20.1±3.2 [20.1]	0.242	0.384
Lipids (%)	26.1±5.1 [26.3]	29.0±6.2 [28.9]	**0.002**	**0.006**
Fibers (g/day)	25.1 (19.2-34.1)	22.6 (15.8-28.9)	0.058	0.355
SFA (%)	7.7±2.6 [7.8]	8.7±3.1 [8.6]	**0.041**	0.462
Polyunsaturated fatty acids (%)	5.3±1.7 [5.4]	5.8±1.7 [5.8]	0.101	0.212
Monounsaturated fatty acids (%)	8.4±2.8 [8.5]	9.2±2.9 [9.2]	0.076	0.148
Cholesterol (mg/day)	260±101 [252]	262±107 [269]	0.894	0.267
Linoleic acid (g/day)	10.8±4.6 [10.4]	11.3±6.3 [11.6]	0.598	0.160
Linolenic acid (g/day)	1.25 (1.01-1.57)	1.35 (1.09-1.67)	0.279	0.061
Oleic acid (g/day)	17.6±6.5 [17.0]	17.5±7.1 [18.1]	0.895	0.288
Calcium (mg/day)	684±255 [668]	673±192 [688]	0.768	0.555
Magnesium (mg/day)	313±96.0 [303]	296±117 [306]	0.326	0.880
Potassium (mg/day)	3315±977 [3204]	2917±848 [3022]	**0.008**	0.075
Sodium (mg/day)	4186±930 [4086]	3932±954 [4027]	0.101	0.619

Quantitative data are reported as means±SD [adjusted mean] or median and percentiles (25th-75th). **Student's *t*-test or Mann-Whitney test (continuous variables with symmetric or asymmetric distribution, respectively). ^#^Adjusted for calorie intake. SFA: saturated fatty acids. Bold type indicates statistically significant.

Nutrients that presented significant association with moderate/severe CAC after being adjusted for risk factors in the multivariate model were higher lipid intake (P=0.005), lower carbohydrate intake (P=0.040), and higher intake of SFA % (P=0.013). A 1% increase in lipid intake entailed a 4% increase in the prevalence of moderate/severe CAC. In addition, a 1% increase in the intake of SFA entailed an 8% increase in the prevalence of the outcome. On the other hand, a 1% increase in carbohydrate intake caused a 2% reduction in the likelihood of moderate/severe CAC, as shown in Table 4.

**Table 4:** Modified Poisson regression to evaluate independently associated dietary-intake factors with moderate/severe coronary artery calcification (CAC).

Variables	Prevalence ratio (95%CI)	P
Lipids (%)	1.04 (1.01-1.07)	**0.005**
SFA (%)	1.08 (1.02-1.14)	**0.013**
Carbohydrates (%)	0.98 (0.96-0.99)	**0.040**
Linolenic acid (g/day)**	1.30 (0.99-1.72)	0.058
Calories (kcal)	1.00 (0.99-1.00)	0.063
Monounsaturated fatty acids (%)	1.06 (0.99-1.12)	0.066
Fibers (g/day)	0.98 (0.97-1.00)	0.093
Cholesterol (mg/day)	1.00 (1.00-1.00)	0.117
Potassium (mg/day)	1.00 (1.00-1.00)	0.124
Oleic acid (g/day)	1.02 (0.99-1.05)	0.199
Sodium (mg/day)	1.00 (1.00-1.00)	0.326
Linoleic acid (g/day)	1.01 (0.99-1.04)	0.393
Polyunsaturated fatty acids (%)	1.04 (0.94-1.15)	0.430
Calcium (mg/day)	1.00 (1.00-1.00)	0.557
Magnesium (mg/day)	1.00 (0.99-1.00)	0.596
Proteins (%)	1.01 (0.96-1.06)	0.660

Adjusted for the following risk factors: age, education level, alcohol intake, waist circumference classification, family history of CAD, high blood pressure, diabetes mellitus, high cholesterol levels, and level of physical activity. **Linolenic acid underwent logarithmic transformation to be entered in the model. SFA: saturated fatty acids. Bold type indicates statistically significant.

## Discussion

In this study, the association between the intake of macronutrients, some macrominerals, and CAC was examined. The results presented in the sample of asymptomatic white men of the Southern region of Brazil showed that only the intake of the macronutrients consisting of lipids, carbohydrates, and SFA was related to CAC, after being adjusted for confounding factors. No association with CAC was observed with other nutrients.

It is difficult to directly compare the results of previous studies with the present study, mainly due to the different methods of each study. Few studies have investigated the association between macronutrient intake and CAC. More recently, two studies found no association between macronutrient intake and CAC (14,15). Only a prospective longitudinal study on the association between the dietary intake of healthy premenopausal women and subsequent postmenopausal subclinical atherosclerosis over an 11-to-14-year follow-up found a positive association between the intake of saturated fat and carbohydrates with CAC (16).

Nutritional habits, particularly the use of dietary fat, are associated with atherosclerosis process (29). Most studies showed that the type and the quality of dietary fat is more important for the development of CVD than the total dietary fat intake (30). In our study, we observed an association between the proportion of total lipid intake and CAC, in which a 1% increase in total lipid intake entailed a 4% increase in prevalence of moderate/severe CAC. Other studies that have investigated the association between macronutrient intake and CAD found no association with the proportion of total lipid intake (14-16). The proportion of total fat intake includes all fat subtypes, and these have different effects on the risk of atherosclerosis (31).

Higher intake of SFA was significantly associated with risk of coronary heart disease (CHD), and this effect can be mediated particularly by an increase of LDL cholesterol (32). The SFA are the dietary factors that impact LDL cholesterol levels the most. A 1% increase in energy from SFA is associated with an LDL cholesterol increase of 0.8-1.6 mg/dL (33). “The Seven Countries Study” conducted by Keys et al. (34) was the first international epidemiological study about CAD to address the role of diet in the development of atherosclerosis. The results showed a positive association between SFA and CAD (35).

A recent study conducted by Guasch-Ferré et al. (36) with 7038 subjects with high risk for CVD of the study “Prevención con Dieta Mediterránea (PREDIMED)” (“Prevention with Mediterranean Diet”) and a 6-year follow-up, showed that SFA is associated with a higher risk for CVD. Comparing extreme quintiles, higher intake of SFA is associated with an 81% higher risk for CVD (HR=1.81; 95%CI: 1.05-3.13).

In the present study, we found an association between the proportion of total SFA intake and CAC, in which a 1% increase in total SFA intake entailed an 8% increase in the prevalence of moderate/severe CAC. Our data are in accordance with those of the study by Park et al. (16), which found a positive association between SFA intake and CAC. SFA is found in different foods of animal origin (e.g., meat, milk, dairy products), tropical oils (e.g., palm, coconut, cocoa butter), and a number of industrialized food (e.g., cookies, cakes, doughnuts, pies) (37). Recent studies that applied computed tomography (CT) to the study of ancient Egyptian mummies showed a high prevalence of calcification in different vascular beds (37,38). Even though the diet of an ancient Egyptian, with or without atherosclerosis, is difficult to ascertain, hieroglyphic inscriptions on the walls of an Egyptian temple indicate that food rich in SFA, such as meat (e.g., bovine, ovine, caprine), aquatic birds, bread, and cake were regularly eaten (38).

Although a recent systematic review and meta-analysis reports no significant association between SFA intake and the risk of CAD development (39), the guidelines of the European Society of Cardiology (ESC), the European Atherosclerosis Society (EAS), and the American Heart Association (AHA) established clear targets about dietary fat intake for the prevention and treatment of cardiovascular diseases (33,40). The intake of SFA should be <10% of the total caloric intake, and should be even less (<7% of energy) in the case of high cholesterol levels.

A higher intake of carbohydrates (a percentage of the total energy intake) has shown a significant protective effect, considering that a 1% increase in carbohydrate intake led to a 2% decrease in the likelihood of moderate/severe CAC. Our results are in line with the findings of the study by Park et al. (16), since carbohydrates have also shown a significant protective effect in CAC development. A possible explanation for this beneficial effect is a higher intake of complex carbohydrates, including whole grains and dietary fibers (32).

Our data showed evidence that national and international guidelines should be complemented, considering the importance of limiting total SFA intake, and increasing high-quality carbohydrate intake for reducing CAD and CAC risk.

Further studies, with a more heterogeneous population in sociodemographic characteristics, are needed to investigate the association among macro- and micronutrient intake with CAC.

The results of the present study showed that a higher intake of total lipids and SFA were associated with higher CAC scores, while a higher intake of carbohydrates over lipids was associated with lower CAC scores in asymptomatic men living in the community.

### Limitations

Our study had some limitations. The number of subjects was small and only healthy white men were included. Because it was a cross-sectional study, it cannot rule out the effect of reverse causality. Additionally, regarding the qualitative aspect of the macronutrient carbohydrate, the types were not individually analyzed (e.g. complex carbohydrates versus simple sugar).
